# Deep-Brain Stimulation of the Subthalamic Nucleus Selectively Decreases Risky Choice in Risk-Preferring Rats

**DOI:** 10.1523/ENEURO.0094-17.2017

**Published:** 2017-08-07

**Authors:** Wendy K. Adams, Cole Vonder Haar, Melanie Tremblay, Paul J. Cocker, Mason M. Silveira, Sukhbir Kaur, Christelle Baunez, Catharine A. Winstanley

**Affiliations:** 1 Department of Psychology, Djavad Mowafaghian Centre for Brain Health, University of British Columbia, Vancouver V6T 1Z3, Canada; 2 Institut de Neurosciences de la Timone, UMR7289 Centre National de la Recherche Scientifique and Aix-Marseille Université, 13005, Marseille, France

**Keywords:** addiction, basal ganglia, decision making, impulsivity, Parkinson’s disease, rat gambling task

## Abstract

Deep brain stimulation of the subthalamic nucleus (STN-DBS) can improve the motor symptoms of Parkinson’s disease (PD) and negate the problematic side effects of dopamine replacement therapy. Although there is concern that STN-DBS may enhance the development of gambling disorder and other impulse control disorders in this patient group, recent data suggest that STN-DBS may actually reduce iatrogenic impulse control disorders, and alleviate obsessive-compulsive disorder (OCD). Here, we sought to determine whether STN-DBS was beneficial or detrimental to performance of the rat gambling task (rGT), a rodent analogue of the Iowa Gambling Task (IGT) used to assess risky decision making clinically. Rats chose between four options associated with different amounts and probabilities of sugar pellet rewards versus timeout punishments. As in the IGT, the optimal approach was to favor options associated with smaller per-trial gains but lower timeout penalties. Once a stable behavioral baseline was established, electrodes were implanted bilaterally into the STN, and the effects of STN-DBS assessed on-task over 10 consecutive sessions using an A-B-A design. STN-DBS did not affect choice in optimal decision makers that correctly favored options associated with smaller per-trial gains but also lower penalties. However, a minority (∼25%) preferred the maladaptive “high-risk, high-reward” options at baseline. STN-DBS significantly and progressively improved choice in these risk-preferring rats. These data support the hypothesis that STN-DBS may be beneficial in ameliorating maladaptive decision making associated with compulsive and addiction disorders.

## Significance Statement

Deep brain stimulation of the subthalamic nucleus (STN-DBS) may constitute a relatively safe and effective alternative to pharmacotherapy, not just for Parkinson’s disease (PD), but for disorders of addiction and compulsion in which decision making is compromised. However, concern remains over whether this manipulation may itself trigger impulse control deficits or risky decision making, as may be predicted from rodent lesion data. Here, we directly test this hypothesis, and evaluate the effects of STN-DBS in rats performing a rodent gambling paradigm based on the Iowa Gambling Task (IGT) used clinically. Far from inducing impulsivity or exacerbating risky choice, STN-DBS selectively improved decision making in animals exhibiting a risk-preferring strategy at baseline. These data suggest that STN-DBS may be beneficial in the treatment of psychiatric, rather than solely neurologic, conditions.

## Introduction

Given the limited efficacy of dopamine replacement therapies (DRT) such as l-DOPA for Parkinson’s disease (PD), deep brain stimulation (DBS) is becoming more common to relieve the motor symptoms of PD (Limousin et al., 1995; Benabid et al., 1998; Martinez-Ramirez and Okun, 2014). In addition, the realization that D_2/3_ family agonists can lead to the *de novo* development of serious impulse control and addiction problems, including gambling disorder (GD), has made neurologists reluctant to prescribe this class of drugs, despite their noted efficacy at restoring motor function (Lawrence et al., 2003; Evans and Lees, 2004; Weintraub et al., 2006; Voon et al., 2011). Both the globus pallidus internal (GPi) and subthalamic nucleus (STN) are common targets for DBS, and can lead to significant improvements in motor function. As DBS involves invasive surgery, it is generally reserved for later-stage PD patients for whom DRT has started to fail, yet there is increasing interest in applying DBS at earlier stages of the disease to maximize therapeutic benefit (Charles et al., 2012; Schuepbach et al., 2013).

STN-DBS can result in a more dramatic reduction in DRT dosing as compared to GPi-DBS (Follett et al., 2010; Odekerken et al., 2013; Liu et al., 2014), therefore this region may be a particularly valid target for those experiencing adverse reactions to DRT. However, the STN influences a wide range of non-motor functions, including impulsivity and decision making (Aron and Poldrack, 2005; Frank, 2006; Ballanger et al., 2009b), and rodent studies have been particularly informative in showing that STN lesions can increase incentive motivation, motor impulsivity, and decrease attention (Baunez et al., 1995; Baunez and Robbins, 1997; Baunez et al., 2002; Winstanley et al., 2005; Uslaner and Robinson, 2006). Furthermore, STN-DBS in PD patients can result in speedier, and arguably less reflective, decision making (Frank, 2006), transiently increase loss-chasing in a gambling simulation (Rogers et al., 2011), and may even precipitate the onset of GD (Witt et al., 2004; Smeding et al., 2007), although support for the latter is somewhat ambivalent (Hälbig et al., 2009; Demetriades et al., 2011; Rodriguez-Oroz et al., 2011). Indeed, a recent meta-analysis comparing DBS of the STN and GPi in PD concluded there were no differences in psychiatric complications resulting from targeting of either area (Wang et al., 2016). The fact that the STN influences cognitive function may, in fact, be of benefit to other patient groups, in that STN-DBS has been suggested as an efficacious treatment for obsessive-compulsive disorder (OCD) and substance use disorder (SUD; Mallet et al., 2008; Pelloux and Baunez, 2013). However, comparatively little is known regarding the effects of STN-DBS on relevant higher-order cognitive processes, particularly in the absence of PD.

The Iowa Gambling Task (IGT), in which subjects aim to maximize money or points through selecting from four decks of cards, was originally developed as a laboratory-based test of “real-world” decision making sensitive to frontal cortex damage (Bechara et al., 1994). The optimal strategy is to avoid “high-risk, high-reward” decks that are paired with disproportionately heavy penalties, and instead favor those associated with incremental yet consistent gains over time. Numerous clinical populations, including SUD, GD, and OCD, exhibit impairments on the IGT, and such persistent choice of the risky options may be a cognitive endophenotype for psychiatric vulnerability (Winstanley and Clark, 2016). To address whether STN-DBS may improve or impair such decision making, we therefore evaluated the effects of this manipulation in rats performing the rat gambling task (rGT), a behavioral paradigm based on the IGT. Given that STN-DBS may specifically impact individuals exhibiting maladaptive choice strategies, we deliberately analyzed whether this manipulation differentially affected optimal decision-makers versus risk-preferring rats.

## Material and Methods

### Subjects

Male Long Evans rats (*n* = 24; initial weight: 250-275 g; Charles River) were pair-housed with free access to water under a reverse light cycle in a climate-controlled colony. Rats were food restricted to 85% of their free-feeding weight and maintained on 14 g of standard rat chow per day plus any sugar pellets earned on task (∼5 g/d). All experimental practices were in accordance with the Canadian Council on Animal Care, and approved by the Animal Care Committee of the University of British Columbia.

### Behavioral apparatus

Preoperative behavioral training took place in 12 standard Med Associates five-hole operant chambers housed in ventilated sound-attenuating cabinets. Each chamber featured a food tray outfitted with both a stimulus light and an infrared beam for detecting nose-poke inputs. Sucrose pellets (45 mg; Bio-Serv) could be delivered to this tray from an external food hopper and a house light was positioned on the chamber wall above. An array of five response apertures was located on the opposite wall, each equipped with stimulus lights and infrared beams for detecting input. Four boxes were equipped with a counterbalanced arm and removable roof insert to enable tethering, and postoperatively only these four boxes were used for behavioral testing. It was possible to deliver bilateral DBS via programmable electrical stimulators to animals within two of these latter boxes at any one time. All of the operant chambers ran according to MedPC (RRID: SCR_014721) programs written by C.A.W. and controlled by an IBM-compatible computer.

### Behavioral training

**Figure 1.**
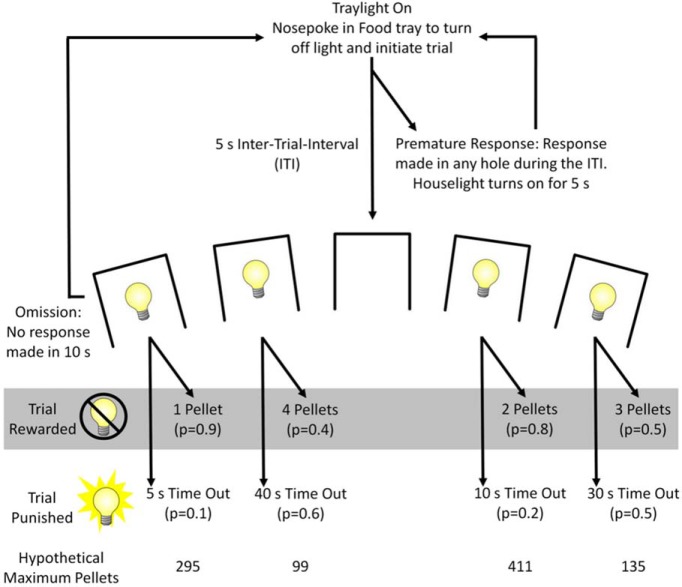
Task schematic of the rGT. A nose poke response in the food tray extinguished the traylight and initiated a new trial. After an ITI of 5 s, four stimulus lights were turned on in holes 1, 2, 4, and 5, each of which was associated with a different number of sugar pellets (one to four pellets, labeled P1-P4). The animal was required to respond at a hole within 10 s. This response was then rewarded or punished depending on the reinforcement schedule for that option (indicated by the probability of a win or loss in brackets). If the animal was rewarded, the stimulus lights turned off and reward was delivered. If the animal “lost,” the stimulus light in the chosen hole flashed at a frequency of 0.5 Hz for the duration of the punishing timeout, and all other lights were extinguished. The order of the options from left to right was counterbalanced across the cohort (version A as shown, version B: 4, 1, 3, 2). The maximum number of pellets available per 30-min session shows that P1 and P2 were better than P3 and P4. The percentage choice of the different options was the primary dependent variable. A score variable was also calculated, as for the IGT, to determine the overall level of risky choice as follows: [(P1 + P2) – (P3 + P4)]. A negative score indicated a preference for the risky options, and rats were categorized as optimal decision-makers or risk-preferring rats accordingly. Adapted with permission from Barrus et al. (2015).

Subjects (*n* = 24) were trained to perform the rat gambling task (rGT) as described previously (Zeeb et al., 2009). In the rGT, subjects sample between four options associated with different magnitudes and probabilities of sugar pellet rewards versus timeout punishments to maximize reward earned. In each 30-min session, subjects chose between one of four options, designated by illumination of response holes 1, 2, 4, and 5 (Fig. 1, task schematic). Each hole was associated with a distinct probability and magnitude of reward or timeout punishments, and are subsequently identified based on the number of potential pellets earned per choice, one pellet (P1), two pellets (P2), three pellets (P3), and four pellets (P4). As in the IGT, the optimal approach in the rGT was to favor options associated with smaller per-trial gains but lower timeout penalties; consistent choice of the smaller reward options was advantageous due to more frequent rewards, but also less frequent and shorter timeouts, with the two-pellet choice (P2) resulting in the most reward earned per unit time.

Subjects began each trial by nose-poking in the illuminated food tray. This response extinguished the tray light and resulted in a 5-s intertrial interval (ITI), during which all lights in the chamber were extinguished. If subjects withheld responding during the ITI, holes 1, 2, 4, and 5 of the array were illuminated for 10 s. A response in any illuminated hole turned off all stimulus lights and led to either onset of the tray light and delivery of a reward, or the start of a timeout “punishment” period. Rewarded trials led to illumination of the food tray and immediate delivery of the appropriate number of sucrose pellets. If a trial was punished, no reward was delivered and the stimulus light within the chosen hole flashed at 0.5 Hz until the punishing timeout elapsed, at which point the tray light was illuminated. If rats failed to make a nose poke in one of the illuminated holes within 10 s, the trial was counted as an omission. Following completion of any trial type, the tray light was reilluminated and subjects could begin a new trial by nose-poking therein. As in the 5-choice serial reaction time task (5CSRT; a rodent analog of the continuous performance test, designed to measure visuospatial attention, motor impulsivity and motivation; Carli et al., 1983; Robbins, 2002), a response made in one of the five response holes during the ITI was punished by a 5 s timeout period and recorded as a premature response. These premature responses serve as a well-validated index of motor impulsivity (Robbins, 2002). The timeout period was marked by illumination of the house light, after which the food tray light was reilluminated and subjects could commence a new trial. Training and testing continued each week day until a statistically stable baseline was observed across all variables measured (44 sessions).

### Electrode implantation

Stimulating electrodes consisted of two platinum wires (EEP-1495 MS303/9-A/SPC Plastics One) sheathed in 9 mm of 22-gauge PEEK tubing, such that 0.5 mm of each electrode was left bare. Animals were anesthetized with 2% isoflurane in oxygen and then secured in a stereotaxic frame. Once anesthetized, animals were given 5 mg/kg ketoprofen subcutaneously. The incisor bar was initially set to -3.3 mm. Great care was taken to ensure the skull was level before implanting the electrodes, and the incisor bar adjusted until there was <0.2 mm difference in height between bregma and lambda. Using standard stereotaxic technique under aseptic conditions, sterile bilateral stimulating electrodes were inserted at an angle of five degrees off vertical through bore holes made in the skull, and then secured with bone screws and dental cement, according to the following stereotaxic coordinates: anteroposterior, -3.8 from bregma; medial/lateral, ±3.1 from the midline; dorsoventral from skull, -8.4. Plastic dust caps were used to protect the external electrode contacts. Animals were given a week to recover in their home cage before behavioral testing resumed. Animals were then reassessed on the rGT until stable baseline performance was observed over a minimum of three consecutive sessions.

### STN-DBS during rGT performance

**Figure 2.**
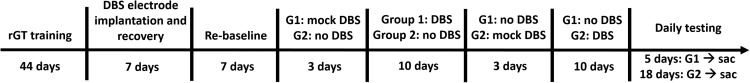
Experimental time line delineating the duration and order of each phase of the study.

A timeline showing the phases of the behavioral experiment is provided in Figure 2. A within-subjects design was used, and all animals experienced STN-DBS. The start of DBS was synchronized to the start of the rGT test session, and remained on throughout the entire 30-min session. Each rat received DBS repeatedly, throughout ten successive, daily rGT test sessions. Animals were divided at random into two groups. Group 1 (G1) received three “mock DBS” rGT sessions, during which the electrodes were connected to the stimulators via spring-loaded tethers, but stimulation was OFF, to acclimatize animals to being tethered. This group then received STN-DBS throughout 10 consecutive rGT sessions, during which the stimulators were turned ON (130 Hz, 60-μs pulse width). Before the first behavioral test session, the current amplitude was adjusted for each individual rat according to former studies (Rouaud et al., 2010), and for each electrode, to just below that which induced the well-known hyperkinetic motor effects in the contralateral paw (treading, “piano playing”). Group 2 (G2) performed the rGT as per baseline training during this period. In each behavioral run of four animals, two therefore received STN-DBS or mock DBS, and two did not. Once group 1 had completed the tenth STN-DBS session, the conditions were switched such that group 2 then received three sessions of mock DBS, followed by ten STB-DBS sessions, and group 1 simply performed the rGT. After five additional behavioral sessions, group 1 was humanely sacrificed (sac) via live decapitation, the brains removed and flash frozen on dry ice, and stored at -80°C. In case any effects of STN-DBS persisted beyond the stimulation epoch itself, care was taken to ensure all rats completed the same number of poststimulation sessions. As such, group 2 performed an additional thirteen rGT sessions before euthanasia, such that data were collected from 18 poststimulation sessions for all rats.

### Histology

The brains were immersion-fixed in 4% paraformaldehyde at 4°C for 7 d, and then transferred to 30% sucrose solution. Tissue was sectioned into 30-µm sections on a cryostat set to -20°C. These slices were mounted onto 2% gelatin-coated slides and stained with cresyl violet. The location of electrode tips were then plotted onto standard rat brain sections (Paxinos and Watson, 1998).

### Data analyses

Data from each experiment were analyzed using SPSS (version 24.0; SPSS). As per previous reports (Zeeb and Winstanley, 2011, 2013; Ferland and Winstanley, 2017), the following rGT variables were analyzed: score (the sum of advantageous choices minus the sum of disadvantageous choices: [P1 + P2]-[P3 + P4]); percentage choice of each option, percentage premature responses, percentage omissions, trials completed, choice- and reward collection latencies. A statistically stable pattern of behavior was verified by a repeated-measures ANOVA across data from at least three consecutive sessions, with session (three levels) and, where appropriate, choice option (four levels: P1-P4) as within-subjects factors, in which the session effects and session × option interactions were not significant. Animals with a mean positive score at baseline were designated as “optimal decision makers,” whereas rats with negative scores were classified as “risk-preferring” (Ferland and Winstanley, 2017). This between-subjects factor (group) was included in all analyses. To determine if risk-preferring rats differed with respect to weight gain as a result of pair-housing, body weight (grams) data over the course of the experiment were analyzed by within-subjects ANOVA with weight record (38 levels) as a within subjects factor, and group as a between-subjects factor.

The effects of mock DBS were also determined by ANOVA, by comparing data from these three sessions to three stable postoperative baseline sessions. Any effect of STN-DBS (sessions 1-3, sessions 4-6) were analyzed through comparison to mock DBS. The final four DBS sessions were similarly compared to the three mock DBS sessions plus the last postoperative baseline session, to correctly populate the ANOVA fields with four sessions in each stimulation condition. Poststimulation sessions were divided into three day bins, and whether any effects of STN-DBS were still evident once stimulation had ceased was again determined via ANOVA through comparison with the three mock DBS sessions. Results are expressed as mean ± SEM. Differences were considered significant at *p* < 0.05.

## Results

### Histology

**Figure 3.**
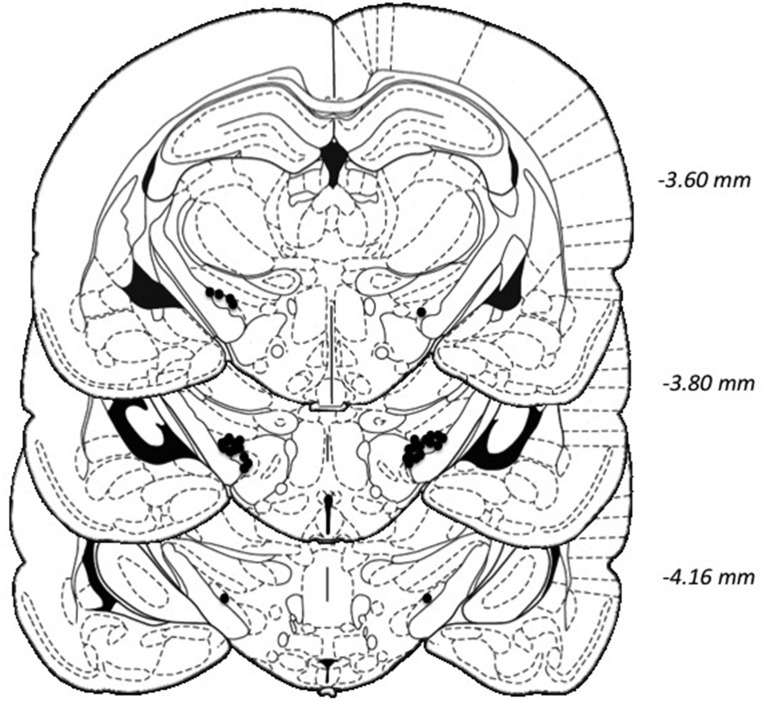
Histologic verification of electrode location. The location of all acceptable electrode tips within the STN are shown in black circles. Coordinates are relative to bregma. Plates modified from Paxinos and Watson (1998).

Data from five rats were excluded from the experiment due to electrode failure (*n* = 1), loss of head-cap (*n* = 2), seizures on stimulation (*n* = 1), or general ill-health (*n* = 1). Two additional rats were excluded from data analyses due to incorrect placement of the electrode, leaving a final *n* = 17 (optimal decision makers: *n* = 13; risk-preferring: *n* = 4). The location of the electrode tips for these 17 subjects is shown in Figure 3.

### Effects of STN-DBS on risky choice

**Figure 4.**
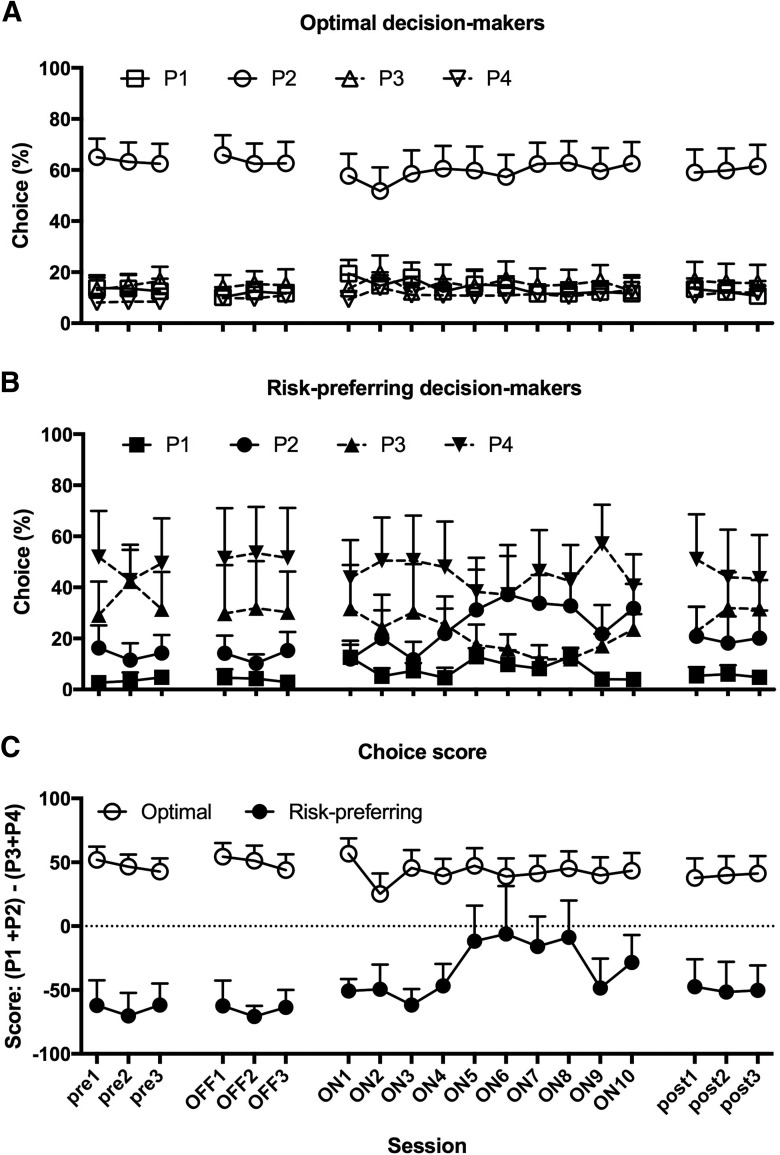
Effects of STN-DBS on decision-making variables. While STN-DBS did not alter relative preference for any of the four options in optimal decision makers (***A***), this manipulation significantly increased optimal choice in risk-preferring rats across the last seven DBS sessions, as indicated by increased choice of the best option, P2 (***B***), and a resulting increase in the score variable (***C***). This effect was no longer evident once stimulation was no longer applied during the rGT. Data shown are group mean ± SEM.

**Figure 5.**
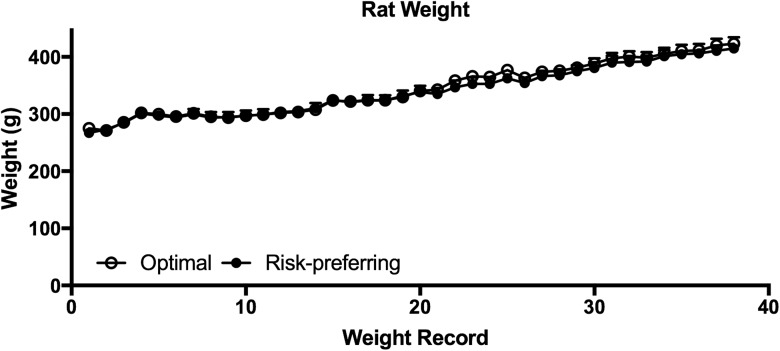
Weight gain throughout food restriction and pair-housing. Body weight was recorded 3–5 d per week during the first month of food restriction, and at least once per week for the duration of the experiment. As expected, all rats showed robust weight gain over time, and the rate or degree of weight gain did not differ between optimal decision-makers and risk-preferring rats. Data shown are group mean ± SEM.

Animals became accustomed to tethering over three mock stimulation sessions before DBS, as indicated by stable patterns of choice across all 4 options (Fig. 4*A*,*B*; session: *F*_(2,30)_ = 2.211, *p* = 0.127; session × risk preference: *F*_(2,30)_ = 0.066, *p* = 0.936), and stable score values across session (Fig. 4*C*; session: *F*_(2,30)_ = 0.697, *p* = 0.506). This mock DBS baseline matched the stable postoperative baseline established following electrode implantation, and is therefore a reliable representation of basal decision-making patterns in these rats (Fig. 4*C*; score, postop/mock: *F*_(1,15)_ = 0.024, p= 0.880; postop/mock × risk preference: *F*_(1,15)_ = 0.001, *p* = 0.976; risk preference: *F*_(1,15)_ = 29.753, *p* = 0.0001). Animals designated as risk-preferring did not differ from optimal decision makers in terms of weight gain during pair-housing, and all animals gained weight robustly during the course of the experiment (Fig. 5; weight record: *F*_(37,555)_ = 153.43, *p* < 0.0001; weight record × risk preference: *F*_(37,555)_ = 0.44, *p* = 0.99; risk preference: *F*_(1,15)_ = 0.11, *p* = 0.75).

Comparing responding during the mock stimulation sessions to the first three DBS sessions, no effects of DBS on choice were apparent in either optimal-decision-makers or risk-preferring rats (Fig. 4; choice: stimulation on/off × risk preference × option: *F*_(3,45)_ = 0.524, *p* = 0.668; score: stimulation on/off × risk preference: *F*_(1,15)_ = 0.622, *p* = 0.44). However, during DBS sessions 4–6, the risk-preferring group selectively and specifically increased choice of the best option, P2 (Fig. 4*B*; stimulation on/off × risk preference × option: *F*_(3,45)_ = 3.775, *p* = 0.017; P2: stimulation on/off × risk preference: *F*_(1,15)_ = 7.476, *p* = 0.015). This change in choice is also reflected in a significant increase in risk-preferring rats’ score (Fig. 4*C*; stimulation on/off × risk preference: *F*_(1,15)_ = 9.426, *p* = 0.008; risk-preferring only: stimulation on/off: *F*_(1,3)_ = 11.216, *p* = 0.044; optimal decision makers only: stimulation on/off: *F*_(1,12)_ = 0.707, *p* = 0.417). This effect persisted through the subsequent DBS sessions (Fig. 4; choice: stimulation on/off × risk preference × option: *F*_(3,45)_ = 2.901, *p* = 0.045; score: stimulation on/off × risk preference: *F*_(1,15)_ = 5.837, *p* = 0.029), but was no longer evident once stimulation was discontinued: risk-preferring rats once again started to make significantly more risky choices in the three poststimulation sessions as compared to on STN-DBS (stimulation on/post × session × risk preference × choice: *F*_(6,90)_ = 2.667, *p* = 0.02), and there was no significant difference in choice patterns when comparing mock stimulation to the first three poststimulation sessions (choice: stimulation off/post × risk preference × choice: *F*_(3,45)_ = 0.612, *p* = 0.610; score: stimulation off/post × risk preference: *F*_(1,15)_ = 1.597, *p* = 0.226).

### Effects of STN-DBS on other variables

**Figure 6.**
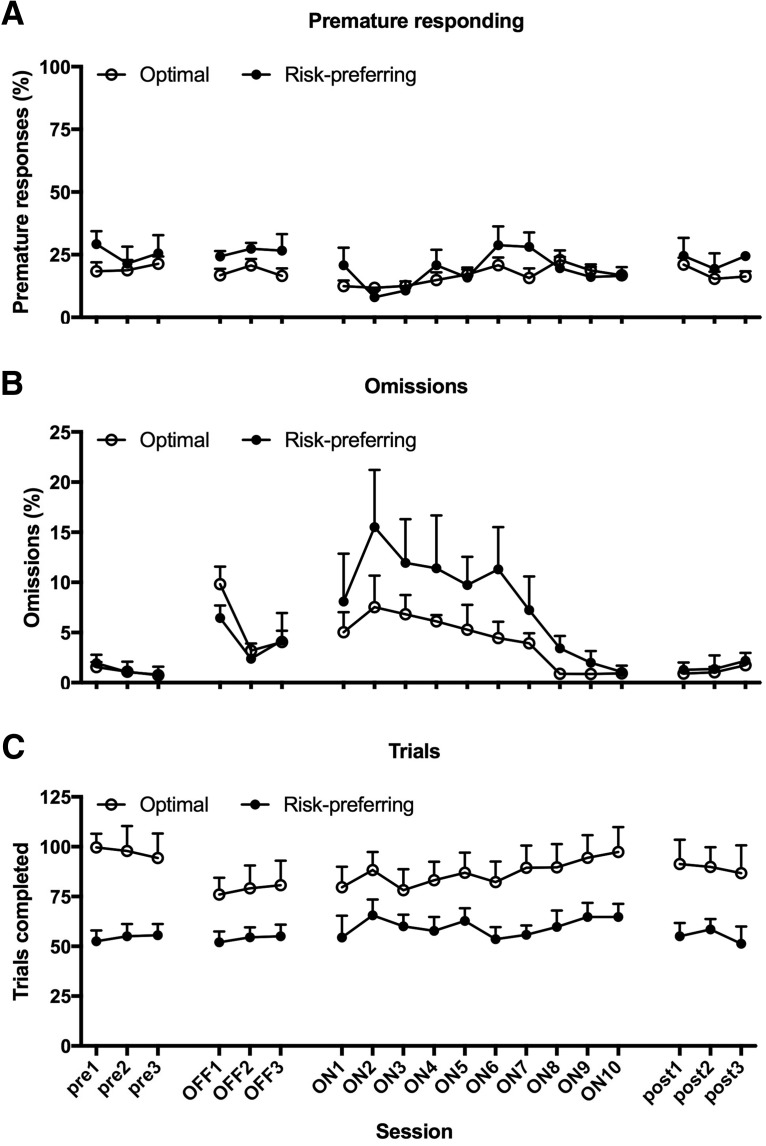
Effects of STN-DBS on non-choice variables. Regardless of baseline decision-making strategy, STN-DBS transiently decreased premature responding, an effect that reached significance in sessions four through six (***A***), and significantly increased omissions (***B***) across all sessions in which stimulation was applied. While STN-DBS did not alter the number of trials completed by optimal decision-makers, risk-preferring rats tended to complete more trials in sessions 4-6 of stimulation (***C***), matching the epoch in which the most dramatic improvements in decision making were observed (Fig. 4). None of these effects were still evident in the three sessions immediately following the final DBS session. Data shown are group mean ± SEM.

Again, all variables measured were stable during the three mock DBS sessions, and this behavioral pattern matched that observed in the postoperative baseline (Fig. 6, Table 1; postop/mock: all *F* ≤ 1.859, *p* ≥ 0.193; postop/mock × risk preference: all *F* ≤ 2.514, *p* ≥ 0.134). Optimal decision-makers and risk-preferring rats did not differ at baseline with respect to the level of premature responses, latency to collect reward, and omissions made (risk preference: all *F* ≤ 0.462, *p* ≥ 0.507). Risk-preferring rats consistently performed fewer trials, as reported previously (Barrus et al., 2015; Ferland and Winstanley, 2017), due to the longer periods of timeout punishment resulting from such a disadvantageous choice strategy (Fig. 6*C*; risk preference: *F*_(1,15)_ = 8.331, *p* = 0.011). Contrary to previous datasets, risk-preferring rats also tended to be slower to make a choice at baseline in the current experiment (Table 1; risk preference: *F* = 6.047, *p* = 0.027).

Comparing the first three DBS sessions to the basal stimulation off condition, there was no effect on premature responding (Fig. 6*A*; stimulation on/off: *F*_(1,15)_ = 0.652, *p* = 0.432; stimulation on/off × risk preference: *F*_(1,15)_ = 1.695, *p* = 0.213). A significant decrease in premature responses was nonetheless evident in the subsequent three DBS sessions, regardless of rats’ basal decision-making strategy (stimulation on/off: *F*_(1,15)_ = 13.560, *p* = 0.002; stimulation on/off × risk preference: *F*_(1,15)_ = 0.338, *p* = 0.569; risk preference: *F*_(1,15)_ = 0.462, *p* = 0.507). This effect was transient, and did not persist across the remaining DBS sessions (stimulation on/off: *F*_(1,15)_ = 1.016, *p* = 0.329; stimulation on/off × risk preference: *F*_(1,15)_ = 0.022, *p* = 0.884; risk preference: *F*_(1,15)_ = 1.028, *p* = 0.327), and poststimulation levels of premature responding were comparable to those observed prestimulation (stimulation off/post: *F*_(1,15)_ = 1.617, *p* = 0.223; stimulation off/post × risk preference: *F*_(1,15)_ = 0.302, *p* = 0.591).

STN-DBS significantly increased omissions in all rats, and this was evident within the first three sessions of stimulation (Fig. 6*B*; stimulation on/off: *F*_(1,15)_ = 12.118, *p* = 0.003; stimulation on/off × risk preference: *F*_(1,15)_ = 0.490, *p* = 0.495) and throughout subsequent sessions (stimulation on/off: all *F* ≥ 29.519, *p* = 0.0001; stimulation on/off × risk preference: all *F* ≤ 2.366, *p* ≥ 0.145). This effect was also temporary, and resolved back to prestimulation levels within the first three sessions of poststimulation testing (stimulation off/post: *F*_(1,15)_ = 1.0, *p* = 0.333; stimulation off/post × risk preference: *F*_(1,15)_ = 0.480, *p* = 0.499). STN-DBS did not affect trials completed in the first three sessions (Fig. 6*C*; stimulation on/off: *F*_(1,15)_ = 2.922, *p* = 0.108; stimulation on/off × risk preference: *F*_(1,15)_ = 2.621, *p* = 0.126; risk preference: *F*_(1,15)_ = 7.103, *p* = 0.018). In the subsequent three DBS sessions, risk-preferring rats tended to increase the number of trials completed, thereby tracking the improvement in optimal choice observed in this subgroup, although they still performed fewer trials than optimal decision makers (stimulation on/off × risk preference: *F*_(1,15)_ = 4.026, *p* = 0.063; risk preference: *F*_(1,15)_ = 6.426, *p* = 0.023). Analyses of subsequent STN-DBS trials revealed no significant difference from mock stimulation, suggesting any effect on trials completed in risk-preferring rats is transient as well as slight (stimulation on/off × risk preference: *F*_(1,15)_ = 0.880, *p* = 0.363; risk preference: *F*_(1,15)_ = 7.770, *p* = 0.014).

STN-DBS also increased choice latency within the first three sessions, an effect that appeared to be more pronounced in optimal decision makers, but was clearly evident in both subgroups of animals (Table 1; stimulation on/off: *F*_(1,15)_ = 17.755, *p* = 0.001; stimulation on/off × risk preference: *F*_(1,15)_ = 3.117, *p* = 0.098; risk preference: *F*_(1,15)_ = 0.101, *p* = 0.754). This effect persisted throughout the remainder of the stimulation sessions (stimulation on/off: all *F* ≥ 29.750, *p* < 0.0001; stimulation on/off × risk preference: all *F* ≤ 0.232, *p* ≥ 0.637). Once again, as soon as stimulation ceased, choice latencies returned to prestimulation levels, although there was no longer a significant difference between optimal decision-makers and risk-preferring rats (stimulation off/post: *F*_(1,15)_ = 2.432, *p* = 0.242; stimulation off/post × risk preference: *F*_(1,15)_ = 0.284 *p* = 0.602; risk preference: *F*_(1,15)_ = 1.701, *p* = 0.212).

**Table 1.** Latencies to make a choice and collect reward during the different phases of the experiment in optimal decision-makers and risk-preferring rats

	Choice latency (s)		Reward collection latency (s)	
Phase	Optimal	Risk preferring	Optimal	Risk preferring
Post-op BL	1.25 (0.18)	1.58 (0.21)	1.56 (0.10)	1.25 (0.17)
Mock DBS	1.27 (0.16)	1.73 (0.14)	1.61 (0.10)	1.33 (0.25)
DBS S1-3	**2.36 (0.22)**	**2.33 (0.16)**	**2.58 (0.35)**	**2.05 (0.29)**
DBS S4-6	**2.16 (0.20)**	**2.81 (0.26)**	**2.38 (0.22)**	**2.46 (0.41)**
DBS S7-10	**2.28 (0.30)**	**2.92 (0.38)**	**2.22 (0.25)**	**2.55 (0.42)**
Post DBS S1-3	1.26 (0.20)	1.79 (0.37)	1.65 (0.10)	1.45 (0.21)

Bold type indicates a significant difference (*p* < 0.05) from the mock DBS sessions. Data are mean (SEM).

Reward collection latency was not significantly different from mock stimulation during the first three sessions of STN-DBS (Table 1; stimulation on/off: *F*_(1,15)_ = 1.85, *p* = 0.194; stimulation on/off × risk preference: *F*_(1,15)_ = 0.288, *p* = 0.60). Similar to choice latency, the time taken to collect reward significantly increased throughout the next three DBS sessions (stimulation on/off: *F*_(1,15)_ = 11.84, *p* = 0.004; stimulation on/off × risk preference: *F*_(1,15)_ = 0.462, *p* = 0.507), and this effect was likewise evident throughout subsequent stimulation sessions (stimulation on/off: *F*_(1,15)_ = 22.67, *p* = 0.0001; stimulation on/off × risk preference: *F*_(1,15)_ = 2.83, *p* = 0.114). Again, once stimulation ceased, performance returned to prestimulation levels (stimulation off/post: *F*_(1,15)_ = 2.23, *p* = 0.16; stimulation off/post × risk preference: *F*_(1,15)_ = 0.001 *p* = 0.99; risk preference: *F*_(1,15)_ = 2.136, *p* = 0.016).

## Discussion

Here, we used a rodent behavioral paradigm with strong translational validity to assess the effects of STN-DBS on risky decision making. We show, for the first time, that STN-DBS selectively improves decision making in animals classified as risk-preferring at baseline, increasing choice of a less risky and more profitable option in these animals. In contrast, optimal decision makers did not alter their choice pattern in response to this manipulation. Although STN-DBS concomitantly increased the time both optimal decision-makers and risk-preferring rats took to make a choice and collect any reward earned, while also increasing the number of omissions made, it did not alter the total number of trials animals completed per session, or increase animals’ tendency to make a premature response. Although not entirely selective for decision making, the effects of STN-DBS on choice in risk-preferring rats are therefore not easily explained as an artifact of universal changes in motivation or locomotion. Indeed, these data suggest that STN-DBS may be a viable alternative to DRT in Parkinsonian patients at risk for impulse control disorders, and may also be an effective treatment for other neurologic or psychiatric conditions hallmarked by risky decision making, such as addiction disorders.

Our data stand in contrast to a previous report which suggested that STN-DBS increased motor impulsivity in rats performing the rGT, while failing to affect decision making (Aleksandrova et al., 2013). Critically, however, STN-DBS did not take place while animals performed the rGT in this former study; instead, stimulation was delivered for two hours before performance of the rGT in an unspecified location. Due to the experimental design employed, it is difficult to determine if the tethering itself contributed to the elevated impulsivity: the animals were not habituated to tethering before the initiation of DBS, and there was no significant difference in the level of impulsive action observed following two hours of tethering, regardless of whether stimulation was on or off. As such, the claim that STN-DBS increases motor impulsivity in this experiment should be interpreted with caution.

Similar to the data reported here, application of STN-DBS during performance of the 5CSRT likewise did not affect premature responding either in rats with 6-hydroxydopamine lesions to the dorsolateral striatum, a model of early PD that results in the loss of dopamine in this area, or in otherwise-intact rats (Baunez et al., 2007). In this latter study, STN-DBS also resulted in slower latencies to respond and increased omissions, again matching the current dataset. Interestingly, in the operant chambers used in this earlier study, animals were required to push a panel inwards to get access to the food tray, and STN-DBS significantly increased the number of such panel pushes. As such, STN-DBS may increase motivation for food reward (Baunez et al., 2005), similar to STN lesions (Rouaud et al., 2010). Although we did not monitor repeated entries to the food tray in the current study, we found that animals were slower to collect the food reward they earned, suggesting animals were not necessarily more motivated to consume the sucrose pellets used as rewards in the rGT. A general boost in motivation is thus unlikely to explain the decrease in risky choice observed in the current study.

This is the first manipulation, to our knowledge, that has selectively improved the maladaptive decision-making pattern of risk-preferring rats in the rGT. The mean score value for these rats increased by around 50 points due to a highly significant and selective increase in choice of the best option, P2. This change in the score variable caused by STN-DBS therefore does not simply reflect random choice among, or indifference to, the various options. These observations may inform our understanding of the neurobiology underlying baseline differences in risky choice. Due to the relatively low incidence of risk-preferring rats, the majority of studies evaluating the impact of neural or pharmacological manipulations have focused instead on modifying choice of optimal decision makers. For example, risky choice can be increased in these animals by lesions to the basolateral amygdala (BLA; Zeeb and Winstanley, 2011), and also by systemic administration of the dopamine D_2/3_ antagonist eticlopride (Zeeb et al., 2009; Zeeb et al., 2013), although this latter effect did not replicate in subsequent studies (Paine et al., 2013; Barrus and Winstanley, 2016). Disrupting connectivity between the orbitofrontal cortex (OFC) and BLA slows learning of the optimal strategy, but silencing the OFC does not affect choice once a stable pattern of decision making has been established (Zeeb and Winstanley, 2011, 2013). Whether aberrant activity in the BLA or OFC or dopamine system contributes to an innate preference for the risky options, or how this could be remedied by STN-DBS, remains unknown.

Indeed, the mechanism through which STN-DBS might precipitate cognitive-behavioral change has yet to be fully clarified. Originally, STN-DBS was thought to normalize aberrant activity in PD patients, locally inhibiting STN function through a depolarization block, thereby restoring motor function in PD through disinhibition of the thalamus (for review, see Gubellini et al., 2009). However, DBS can also excite proximal fibers of passage, resulting in enhanced neuronal firing in terminal regions, as well as increasing the firing of afferent projections (Vitek, 2002; McCracken and Grace, 2007; Gradinaru et al., 2009). The STN is highly interconnected with the ventral pallidum, and thereby receives considerable indirect input from numerous limbic structures, but the STN also receives direct input from the OFC and medial prefrontal cortex (Maurice et al., 1998; Haynes and Haber, 2013). It is theoretically possible, therefore, that STN-DBS may exert beneficial effects on risky choice in the rGT through altering activity in these cortical regions (Ballanger et al., 2009a), a conclusion that would fit with the known contribution of these areas to choice on-task (Paine et al., 2013; Zeeb and Winstanley, 2013; Zeeb et al., 2015).

The beneficial effects of STN-DBS were delayed, in that we did not observe significant improvement in decision making within the first three treatment sessions. Similar effects have been observed with regards to the effects of STN-DBS on attention or motivation, when significant effects could only be obtained after multiple daily sessions (Baunez et al., 2007; Rouaud et al., 2010). Although this time course may suggest that STN-DBS does not reliably induce changes in decision making, the need for repeated stimulation could also be required to trigger neuroplasticity which then results in cognitive change. In support of the latter, previous data suggest that chronic (5 d) of STN-DBS results in dramatic inhibition of glutamatergic synaptic transmission in the striatum, potentially through decreased expression of AMPA and NMDA receptor expression or sensitivity (Gubellini et al., 2006). Such changes may reflect neuroplastic processes, such as long-term depression, that could arise through circuit-level changes in the thalamo-cortico-striatal loop as a result of repeated STN-DBS (Albin et al., 1989). Chronic STN-DBS can also increase expression of brain-derived neurotrophic factor (BDNF) in the striatum (Spieles-Engemann et al., 2011). Whether such neuroplastic events could contribute to new learning in risk-preferring rats, and therefore a shift toward more optimal decision making, remains to be empirically determined. Interestingly, DBS of the fornix increases BDNF levels in the hippocampus (Gondard et al., 2015), an effect which may be linked to the ability of this manipulation to improve memory function in an experimental model of dementia (Hescham et al., 2013).

STN-DBS has been found effective in the treatment of OCD (Mallet et al., 2002; Mallet et al., 2008), a patient group that is likewise impaired on the IGT (Cavedini et al., 2010; Starcke et al., 2010; Zhang et al., 2015), and in which OFC dysfunction has long been documented (Modell et al., 1989; Saxena et al., 1999; Evans et al., 2004). Although therapeutic benefit may still be attributed to normalized frontal function (Le Jeune et al., 2010), recent work has documented an electrophysiological signature associated with OCD in the ventromedial STN that is associated with OCD symptom severity, as well as clinical benefit post-DBS (Piallat et al., 2011; Welter et al., 2011; Mulders et al., 2016). Disrupted information processing at the level of the STN, and its normalization, may therefore play a more fundamental role in both the manifestation of OCD and its resolution following STN-DBS (Mulders et al., 2016). Whether a similar aberration in activity likewise characterizes, and even drives, maladaptive risky choice or compulsive gambling behavior remains to be determined. Based on the current data, and observations that STN-DBS may be beneficial in reducing compulsive drug use (Pelloux and Baunez, 2013), such a possibility may be worthy of investigation.

With regards to addiction risk, some data suggest that patients who have a family history of alcohol use, and are potentially more vulnerable to addictions themselves, are more likely to develop impulse control problems following DRT (Voon et al., 2007). Interestingly, risk-preferring rats, i.e. those who benefited from STN-DBS in the current study, are uniquely and negatively affected by concurrent cocaine self-administration: their decision making becomes even more risky, whereas optimal decision makers are unaffected, despite self-administering comparable amounts of cocaine (Ferland and Winstanley, 2017). Risk-preferring animals also show enhanced cue-induced drug-seeking with extended time in withdrawal, a putative behavioral marker of enhanced relapse vulnerability (Grimm et al., 2001). As such, risk-preferring rats may constitute a useful model of addiction vulnerability. In humans, the time course across which risky decision making is evident in SUD tracks subjective craving for drug, such that both resolve at a similar time point following successful treatment (Wang et al., 2013). Conversely, unresolved and persistent risky decision making on the IGT in SUD patients is a strong predictor of treatment failure (Stevens et al., 2013). As such, a manipulation that ameliorates risky choice on the rGT/IGT, such as STN-DBS, could have therapeutic efficacy for treating SUD. Indeed, a growing body of preclinical and clinical data suggest that STN-DBS may be useful in reducing drug use and drug craving (Baunez et al., 2005; Rouaud et al., 2010; Pelloux and Baunez, 2013). Should our observation that STN-DBS improves risky decision making translate to human subjects, this would suggest an additional cognitive mechanism by which this manipulation improves treatment outcomes.

To conclude, DBS is increasingly being considered for psychiatric conditions, rather than purely neurologic diseases, due to the urgent need for effective treatments, and lack of innovation in effective pharmacotherapeutics for mental illness. Given the invasive nature of the procedure, a cautious approach is understandable, and the need for further research into the potential cognitive effects of DBS, and its mechanism of actions, should not be minimized. However, by making use of the considerable degree of experimental control possible when using an animal model, this study strongly suggests that STN-DBS does not impair decision making under uncertainty, despite fears to the contrary, but may actually ameliorate the maladaptive persistence in the selection of risky options seen in a subgroup of individuals. These data add to the growing body of research arguing that STN-DBS may be effective more widely in disorders of compulsion and maladaptive drive.

## Synthesis

Reviewing Editor: Karen Szumlinski, University of California at Santa Barbara Decisions are customarily a result of the Reviewing Editor and the peer reviewers coming together and discussing their recommendations until a consensus is reached. When revisions are invited, a fact-based synthesis statement explaining their decision and outlining what is needed to prepare a revision will be listed below. The following reviewer(s) agreed to reveal their identity: Erin Calipari.

In consultation with two expert reviewers, I have reached the decision of “Major Revision” regarding your article submitted to eNeuro. Both reviewers and I felt that the article presents new and interesting data that would be of high relevance to the readers of our journal, a few concerns were raised by Reviewer #1 that influence the interpretation of the results. As such, both reviewers and I would like to see these concerns addressed in a revision. These concerns are the following:

1) On page 6, line 106, it is mentioned that rats were pair-housed with food restriction. It is highly questionable that a pair of rats in each cage were equally food restricted, as rodents are socially hierarchical animals, with a dominant would get more foods than a subordinate. Such disproportional food restrictions between a pair of rats in each cage may have affected performance of the gambling task. In order to resolve this issue, we suggest that the authors provide data pertaining to the weight of the rats to confirm the efficacy of the food restriction.

2) A protocol for the deep brain stimulation is insufficiently described on page 9. Was stimulation given 10 times to the same rats repeatedly, with a set of stimulation on each trial for 10 trials? How long before starting each session was stimulation given? A set of stimulation consisted of 130 Hz bursts, but how long was the burst? Providing greater detail pertaining to the specific parameters of the stimulation should resolve this issue.

3) Although the authors have argued that deep brain stimulation decreased risky choices in risk preferring rats, what Figure 4B&C actually shows is that only less than a half of sessions that deep brain stimulation decreased risky choices, and increased optimal choices in these rats. In Figure 4C, it can be seen that choice scores for the first 4 sessions with deep brain stimulation (ON1-4) in risk-preferring rats are apparently not much different from pre, OFF, and post sessions. The fact that the rats require at least 4 sessions of stimulation is very interesting to all of us but requires some deeper discussion. Is the DBS not effective all of the time or under certain circumstances or does it simply take repeated stimulation in order to observe an effect (implying that some latent neuroadaptation is being produced by the stimulation)? Either way, it would be good to speculate on what each alternate possibility might mean for the clinical treatment setting.

As these concerns are likely to require less than 2 months to address, I welcome the receipt of your revised manuscript to eNeuro.
